# Kenyan health stakeholder views on individual consent, general notification and governance processes for the re-use of hospital inpatient data to support learning on healthcare systems

**DOI:** 10.1186/s12910-018-0343-9

**Published:** 2019-01-08

**Authors:** Daniel Mbuthia, Sassy Molyneux, Maureen Njue, Salim Mwalukore, Vicki Marsh

**Affiliations:** 1 Ujamaa Africa, Kenya, 5th Floor, Landmark Plaza, Kamunde Rd, Nairobi, Kenya; 2 KEMRI Wellcome Trust Research Programme, Kilifi, 80108 Kenya; 3 Centre for Tropical Medicine and Global Health, Nuffield Department of Medicine, South Parks Road, Oxford University, Oxford, OX1 3SY UK; 4 Institute for Tropical Medicine, Kronenburgstraat 43, 2000 Antwerpen, Belgium

**Keywords:** Africa, Kenya, Learning healthcare systems, Comparative effectiveness research, Electronic health records, Quality improvement research, Informed consent, General notification, Public engagement, Acceptability, Trust

## Abstract

**Background:**

Increasing adoption of electronic health records in hospitals provides new opportunities for patient data to support public health advances. Such learning healthcare models have generated ethical debate in high-income countries, including on the role of patient and public consent and engagement. Increasing use of electronic health records in low-middle income countries offers important potential to fast-track healthcare improvements in these settings, where a disproportionate burden of global morbidity occurs. Core ethical issues have been raised around the role and form of information sharing processes for learning healthcare systems, including individual consent and individual and public general notification processes, but little research has focused on this perspective in low-middle income countries.

**Methods:**

We conducted a qualitative study on the role of information sharing and governance processes for inpatient data re-use, using in-depth interviews with 34 health stakeholders at two public hospitals on the Kenyan coast, including health managers, providers and researchers. Data were collected between March and July 2016 and analysed using a framework approach, with Nvivo 10 software to support data management.

**Results:**

Most forms of clinical data re-use were seen as an important public health good. Individual consent and general notification processes were often argued as important, but contingent on interrelated influences of the type of data, use and secondary user. Underlying concerns were linked to issues of patient privacy and autonomy; perceived risks to trust in health systems; and fairness in how data would be used, particularly for non-public sector re-users. Support for engagement often turned on the anticipated outcomes of information-sharing processes, as building or undermining trust in healthcare systems.

**Conclusions:**

As reported in high income countries, learning healthcare systems in low-middle counties may generate a core ethical tension between supporting a public good and respecting patient autonomy and privacy, with the maintenance of public trust acting as a core requirement. While more evidence is needed on patient and public perspectives on learning healthcare activities, greater collaboration between public health and research governance systems is likely to support the development of efficient and locally responsive learning healthcare activities in LMICs.

**Electronic supplementary material:**

The online version of this article (10.1186/s12910-018-0343-9) contains supplementary material, which is available to authorized users.

## Background

In spite of progress, the 2017 World Health Report identifies continuing major healthcare and healthcare systems challenges globally, with a disproportionate burden in low-income and low-middle income countries (LMICs) [1]. An innovative approach proposed for expediting progress in healthcare delivery globally, and more recently in LMICs, draws on the increased use of electronic health records (EHR) in health care facilities to inform systematic analyses of the effectiveness of existing care. In this way, the digitization of healthcare has opened up new ways in which learning activities including audits, evaluation and research can be embedded within clinical practice [2, 3].

An approach to learning about healthcare and healthcare systems using existing EHRs is a core characteristic of what has been described as a Learning Healthcare System (LHS), that is, a system ‘designed to generate and apply the best evidence for the collaborative healthcare choices of each patient and provider; to drive the process of discovery as a natural outgrowth of patient care; and to ensure innovation, quality, safety, and value in health care’ [4] . The concept of a LHS embraces many different manifestations, operating at different scales, rather than a uniform approach. To date, experience and debate on the opportunities and challenges of learning healthcare models have focused on high-income countries where EHRs are routinely in place. However, the increasing use of EHRs in public health facilities in LMICs provides an important opportunity to draw on the learning healthcare model to ‘fast track’ improvements in healthcare and systems [2].

In Kenya in particular, the Ministry of Health has been working with different partners to drive the adoption of EHRs in most public hospitals [5]. This potential underlines the need for research on the wider social acceptability of learning healthcare models and the role of information sharing, including general notification and individual consent processes, as a component of this acceptability in LMICs. The study reported in this paper aimed to contribute to the literature through a focus on the ethics of patient and public information sharing and governance for LHS in LMICs, to inform local policy development and feed into the wider international debate. These issues hinge on debate around the definitions applied to the ways that EHRs might be re-used, for example, between audit, evaluation and research, as discussed in the following paragraphs.

### A continuum within learning healthcare system approaches

A core conceptual and practical challenge for learning healthcare models is how to distinguish forms of healthcare that include learning from more traditional types of health research. This is an important ethical and practical question, particularly in establishing how patients’ rights and interests can be respected in a LHS where traditional research governance processes may not be involved [6–8]. The underlying question in these debates concerns how to determine ethically important distinctions between healthcare activities classed as ‘quality improvement’ (QI) and those described as forms of research under a learning healthcare model, including quality improvement research (QIR), comparative effectiveness research (CER) and pragmatic clinical trials (PCT). QI processes are seen as routine and as a basic requirement for effective and accountable health care governance, with no need for specific ethics oversight. QIR, in contrast, includes characteristics more typical of traditional research, such as the production of generalizable new knowledge and use of systematic methods, and is currently often seen to require independent ethics oversight. Typical QIR might include evaluation activities such as a review of medical records or interviews with stakeholders to identify successes and challenges for a particular form of health care, and propose solutions [8].

It has been widely argued that QI and QIR processes are not easily distinguished on the basis of the characteristics described above, and that the implications for governance in shifting from one to the other presents dramatic bureaucratic barriers for important forms of QIR [6, 8, 9]. Similarly, QIR processes are not easy to distinguish from comparative effectiveness research (CER), in which different standard healthcare or healthcare system interventions are systematically compared to assess their relative effectiveness and inform policies going forwards [10].

Within the CER model, some forms of research can be described as pragmatic, linked to their nature as ‘real world’ comparisons of existing interventions, in contrast to more typically research-based clinical trials that are exploratory in nature, and set out to determine impact by discounting real world effects. Even then, the shift from pragmatic trial to exploratory clinical trial is not clear cut but has been described as varying over a range of different parameters from ‘more pragmatic’ to ‘more exploratory’ [11, 12]. Particular advantages noted for QIR, CER and some forms of pragmatic clinical trials are the capacity to respond more quickly to gaps in the existing evidence base around health care provision, including through greater translational benefits, expedited processes and lower costs than are generally the case for traditional research approaches [13–16].

Given these ‘grey areas’ at the boundaries between categories of QI, QIR, CER/pragmatic clinical trials and arguably exploratory clinical trials, our research included consideration of different data re-use scenarios, aiming to contribute to understanding the influence of this shift on participants’ views on the importance of patient and public engagement in our setting.

### Information sharing as an ethical issue for learning healthcare system approaches

As the preceding paragraphs imply, a number of ethical concerns have been raised around the concept of a LHS [17–23], and summarized by Sugarman and Califf (2014) [10]. Two issues are particularly prominent in the literature, and have informed the study described in this paper. The first is the question of what forms of patient and public information sharing are needed for the re-use of clinical data, including individual patient notification or informed consent and general (hospital user or public) notification. For example, requiring informed consent from patients for EHR re-use may generate extra cost and time burdens and can generate important forms of selection bias that undermine science in the learning healthcare model. On the other hand, re-use of individual EHRs without patient knowledge and agreement risks undermining individual autonomy [15, 24–26]. The second issue concerns the forms of governance required around learning healthcare approaches, particularly where individual informed consent might not be sought. There are concerns that bureaucratic delays often involved in ethics review processes may be incompatible with the concept of a continuous learning process [25]. In fact, ethics committee members have described their own lack of clarity about how to assess the ethical issues raised by different forms of CER [18, 27]. Relatedly, there is strong recognition of the need for social accountability in relation to learning healthcare models [28]. As a result, there is a significant literature from high-income countries examining public views on the importance of patient consent and governance for learning healthcare models [22, 29–31]. To the best of our knowledge, no studies have addressed this topic in an LMIC context.

Given the potential of learning healthcare models in LMICs, particularly in countries like Kenya where EHRs are widely in place, this paper reports on a preliminary qualitative study designed to explore the views of health managers, providers and researchers at two county-level hospitals in coastal Kenya on questions on individual and public information sharing (including consent and notification processes) and governance for the re-use of EHRs for different learning purposes. Through this work, and acknowledging the absence of patient, hospital users or general public voices, we aim to support policy development in this and other similar LMIC settings, and contribute to the wider debate in the literature on the role of patient and public engagement in learning healthcare models globally.

## Methods

### Study setting and site selection

The study was conducted in two public hospitals in Kilifi County on the Kenyan coast; Kilifi County (KCH) and Malindi Sub-county Hospitals (MSCH). KCH is the site of the main hub of the KEMRI Wellcome Trust Research programme (KWTRP), an international collaborative research programme working in close collaboration with the Ministry of Health at national and county levels [32]. KWTRP researchers have run many clinical research studies within KCH since its inception in 1989, and supports the county health team to maintain EHRs for all routine paediatric and some adult hospital admissions. EHR data support public health monitoring and research activities, the latter with the consent of individual patients or legal guardians. KWTRP researchers also run studies at MSCH, although at much lower levels of activity. Research governance processes at KWTRP function under the national Kenyan Medical Research Institute (KEMRI) Science and Ethics Review Unit. Since devolution of most government departments to county level in 2013, a Kilifi County Research Office provides oversight of all research conducted in the county, with support from County and Sub-county Hospital Management Committees.

Selection of KCH and MSCH as study sites was on the basis of their experience using clinical data use for audits, service evaluation and research, and, given the preliminary nature of this study, pragmatic considerations of accessibility and the research team being embedded within these hospitals.

### Study participants

We purposively selected and invited 34 healthcare managers, health providers and researchers from KCH and MSCH to participate in this study, based on i) their involvement in the collection, management and use of clinical data for a range of purposes including routine audit, evaluation and comparative effectiveness research; and ii) generating variation in types and levels of professional experience and gender. A summary of participant characteristics is given in Table 1.

**Table 1:** Summary of participant characteristics

Location	Role	Total (34)	Women (17)	Highest qualification	Nationality
Kilifi	Researchers	8	4	Diploma × 2; MSc × 2; PhD × 4	Kenyan 6 UK 1
	Health providers	9	6	BSc ×1; Diploma × 6; MSc × 2	Kenyan 9
	Health managers	7	3	Diploma ×6; MSc ×1	Kenyan 7
Malindi	Researchers	2	0	MSc ×1; Diploma × 1	Kenyan 2
	Health providers	3	2	BSc ×1; Diploma ×2	Kenyan 2
	Health managers	5	2	MSc ×1; Diploma ×2; BSc × 2	Kenyan 5

### Data collection methods

We developed and piloted a set of four scenarios for this study, to represent increasing levels of ‘research’ along a health care-research continuum. These scenarios included routine monthly audits (for example, monthly aggregated data on patient diagnoses are routinely reported to the national government on a statutory basis across Kenyan public hospitals); an evaluation of a treatment guideline; and two types of comparative effectiveness research (CER), including a retrospective comparison of two antibiotics in routine use and a prospective comparison including randomization of patients to one of the two antibiotics under study. The four scenarios are described in detail in Table 2.

**Table 2:** Scenarios used to facilitate discussions

Scenario 1: Use for routine audit	
Clinical hospital data (with people’s names taken off) being used by the County Health Team to assess and report on patterns of different diseases at different times, such as the number of people admitted to hospital with malaria in a given time period.	
Scenario 2: Use for evaluations	
A public health manager in the County Health Team uses clinical and laboratory data from individual patients who have been treated for malaria in hospital (with names taken off) to evaluate whether new guidelines that have been introduced for the in-patient treatment of malaria are improving clinical outcomes overall and over time.	
Scenario 3: CER: Non-randomized Pragmatic Clinical Trials	
It is common in medical practice that there are several different treatments available to treat a given condition, without clear evidence that one treatment works better than the other. For example, many different antibiotics are recommended to treat particular infections, like boils, ear infections or lung infections. In this situation, doctors tend to choose a treatment based on their own or their patients’ personal experiences/preferences. If there was more evidence about which treatments work best and in which situations, both patients and doctors would benefit. One way for researchers to do this is to compare routine clinical data on patient outcomes (e.g. how quickly or completely patients got better after being treated by one drug compared to another). In this kind of research, the researchers DON’T introduce anything different to the normal practice. They only analyze clinical data from patients who were treated to compare the effectiveness of different antibiotics used.	
Scenario 4: CER: Randomized Pragmatic Clinical Trials	
There are many different antibiotics currently approved and used routinely for treating pneumonia. For some of these antibiotics, it’s not known if they work better than others available. For example, let’s think about two such treatments, and call them antibiotic X and antibiotic Y. Both are already approved drugs and are in use at the moment. They are given in similar ways and have similar types and risks of any side effects or more serious reactions. (Serious reactions are very rare). It is therefore unlikely that patients or physicians would have a personal preference for one drug over the other. To find out if there are any differences between these treatments, researchers can ask physicians to prescribe one of these drugs based on a system of chance, and observe over time how well patients respond to the treatments. Over time, the outcomes of patients being treated with one of these two antibiotics can be compared to learn which treatment works best. Once this is known, all the patients can be given the option to change to that treatment.	

In-depth interviews were conducted between March and July 2017, including 24 participants in Kilifi and 10 in Malindi, and lasting between one and two hours. For each of the scenarios described above, we used a set of specific probes to vary context in the following ways: i) changing the type of stakeholders re-using data; ii) asking about data with potentially differing sensitivities; and iii) probing on different forms of engagement, including individual patient consent and notification (without consent) and public notification processes. Interview guides are included as an additional file (Additional file 1). All the interviews were conducted in English, and were audio recorded and transcribed prior to analysis.

### Data management and analysis

Interview summaries were developed early during data collection to allow for immersion in the data. We used a framework approach to analyze our data, involving a systematic process of familiarization, identifying a thematic framework, indexing, charting, mapping and interpretation [33]. The analysis process drew on both deductive and inductive approaches, that is, through deductively following themes explored in interview guides and inductively responding to new or emergent issues raised by participants around the broad topic of enquiry. Coding and data management was done using QSR Nvivo 10. Two researchers (DM and VM) independently coded and developed the initial coding framework. Interview summaries and coded data were brought together to develop final analysis charts as an iterative process to make sense, for example, of individual participants’ changes of opinion over time and allow data to be collated across the major themes and types of participants. DM, VM and SMo undertook the analysis and interpretation of data.

## Results

Across the study, interviews with stakeholders generated complex and in-depth discussions. Complexity was linked to the nature of the scenarios themselves and to the range of influences or conditions underpinning views. Since scenarios were sometimes unfamiliar to participants, and given the inevitable ‘grey areas’ between scenarios, the shift from one data re-use situation to another often had to be highlighted and re-explained to participants. Complexity was also observed in the depth of deliberation emerging during discussions, including weighing up different influences on views. Both forms of complexity led to active reflection and often to changes of opinion during interviews.

In the following sections, we first describe the major arguments raised in relation to the value of sharing information on re-use of EHRs, including as individual consent and general notification processes, across all four scenarios (sections A and B below). These arguments ‘for’ and ‘against’ sharing information are summarized in Table 3. We then go on to show the way that these arguments were drawn upon within each of the scenarios used, highlighting shifts in the way issues were balanced in relation to different forms of data re-use and the form of information sharing that was therefore seen as important (section C). In the final section (D), we describe views on governance across all forms of EHR re-use; showing that these opinions rested on an assessment of who was leading the activity and the main purpose of the data re-use.

**Table 3:** Summary of the main emerging arguments for and against sharing information about clinical data re-use with patients and the public

‘Sharing information is important’	‘Sharing information is not (so) important’
Ownership/rights/trust in patient-physician relationships • Individuals have a right to information e.g. as provided in the hospital service charter • Existing nature of patient-physician relations is based on assumption that data are only used for patient care • Rights persist even if data is de-identified Demonstrating respect to patients • As a way of respecting patients’ autonomy and preferences Openness/nothing to hide/trust & accountability • Important to demonstrate openness/nothing to hide • Will ensure that better quality/more complete data available • Wil provide reassurance/strengthen accountability for governance & of data re-use • Patients will ‘feel good’ Building trust in health system • Increased accountability will build trust in public health facilities & systems • Will create partnership/sense of responsibility towards public health system Normalizing clinical data re-use • Increased acceptability of clinical data re-use over time • Less resources needed for communication over time Reduce risks of harm where data may be sensitive • More important that patients know about data re-use if these may be ‘sensitive’ • Should be able to refuse re-use • Can help to determine whether sensitive or not, since likely context related Change in physician- patient relationship • Physician no longer acting solely in patients’ interest, therefore important for patients to know • Patients actively being involved in research as subjects	Data must be used for public good • Used for planning and public health good, which outweighs individual rights to awareness/consent Impact of refusals on public health planning • Information sharing risks opt-out or refusals of clinical data re-use, which would importantly undermine public health services • Particularly risky for public forms of engagement • Likely to refuse where misconceptions /rumors arise about how data will be used Extra resources needed in already overstretched systems/communication challenges • Overstretched resources put under increased pressure through extra communication needs, risk undermining services • May be very difficult to explain e.g. de-identification & future unknown research • Consent may be meaningless in contexts of unmet health needs/asking patients for permission in resource limited settings might be perceived as coercive Misunderstandings could impact services and trust in the health system • Loss of trust may lead patients to withhold clinical information, refuse routine clinical tests/procedures and avoid care • Misunderstanding / misconceptions could lead to loss of trust in public health systems De-identified/aggregated data re-use not risky • Minimal risk to patients in re-using aggregated/ de-identified data therefore patient engagement not seen as important. • Examples of aggregated data already placed on hospital notice boards Implicit consent already given by patients • In choosing to attend public hospitals, patients give an implicit consent for their data to be re-used

### Arguments for sharing information on clinical data re-use with patients or publics

Most participants consistently felt that it would be important to explain to individual patients that their routine clinical data might be used in a number of ways beyond their own care. This position was underpinned by a number of interrelated arguments, including around an individual’s rights to know; trust in the physician-patient relationship; a value of openness and accountability; and the perceived sensitivity of data. While these arguments are presented separately in the sections following, they often flowed into each other during discussions, and were drawn upon across all scenarios.

#### Individual rights to know

Regarding informing individual patients about clinical data re-use, a common and strongly held argument drew on ideas of an individuals’ rights to know. These arguments were particularly made by providers and researchers, and across all the scenarios:

*‘I do have a right for that [information on data re-use]… so I think everyone else should have that right, sometimes people are not aware even they have these rights … so maybe we are just using that ignorance of these patients which is not good, so I think they should know’ (P14 Kilifi health provider).*

On this account, clinical data were seen as belonging to the person they derived from, giving patients a right to know how their data were being used now and in future. One provider further supported this position by referring to the general right to information enshrined in the government hospital charter, which is publicly displayed on hospital walls. That clinical data were seen as confidential (as described later) added to the sense that patients should be made aware of any such practices.

This argument did not always hinge on whether or not data might be linked to identifiable individuals, with many participants feeling that patients’ rights to know would still pertain where data were de-identified:

*‘Ok... although we are not using the names and locations and the things but still we are using their information, so I think ethically they have the right to know that there is this information is going to be used somewhere else.’ (P14 Kilifi health provider).*

#### Trust in the physician-patient relationship

Some providers and hospital managers linked the need to explain clinical data re-use to the existing nature of physician-patient relationships. This relationship was seen as founded on an understanding, and therefore trust, that a physician would use individual clinical data *only* within that patient’s care.

*‘They should be made aware because...when you talk to patients, they tell you everything because they trust you … so if at the end of the day, that report is going to be used to benefit him or somebody else then he should know that, I think it’s fair enough.’ (P16 Kilifi health provider).*

#### Openness, nothing to hide and building trust and accountability in systems

Many participants saw a fundamental value in openness (in sharing information on clinical data re-use with patients and publics) as generating a better understanding of the value of EHRs for public health planning and as demonstrating that there was ‘nothing to hide’:

*I think they have the right to know... Why would you want to hide things? Because that to me, it’s hiding information from patients (P02 Kilifi provider).*

Openness around clinical data re-use was also seen as a means of showing appropriate levels of respect towards patients and of building trust in the health facility and the wider health system. Here, many participants felt that patients would have more confidence in the health system if they understood that their data were used beyond their treatment to strengthen health systems. In addition, this knowledge was seen as likely to encourage patients to give more accurate information when they come for care, leading to improved quality of data collected in hospitals. This was sometimes the case even for sensitive data.

*I personally, I have had cases where patients refuse to give you some details [For example] they are afraid of sharing their age because according to our culture here when … people know that you are old … something bad might happen to you. ‘I came here for treatment, why do you want my age?” (P05 Kilifi health providers).*

Conversely, failing to share information on clinical data re-use was seen as potentially undermining trust in very fundamental ways, if this was later found out. This concern particularly underpinned arguments on the importance of showing that there is ‘nothing to hide’.

Further related arguments for explaining clinical data re-use to patients were that this would generate a sense of partnership or responsibility towards the health system, that knowing about the existence of a systematic data-use process to build systems would provide reassurance about the levels of oversight and protection in place, and that knowing about the contribution of individual patients to public health would bring personal satisfaction.

*So, I want to know so that I know I’m being protected at a certain level and I can take someone to task in the event that anything wrong happens, you know...’ (P09 Kilifi healthcare manager).*

*If they are asked and they give … all the information they have … they’ll feel happy that … somebody else has gained from it’ (P19 Malindi health provider).*

Further, and in the longer term, openness about clinical data re-use was argued as contributing to a process of normalizing understanding of this use that would reduce sensitivities in future.

#### Sensitivity around types of data

Arguments for sharing information on data re-use were made more strongly and commonly where data were seen as sensitive. Here, the form of information sharing needed was often described as ‘seeking permission’ rather than ‘general notification’. However, it was not easy for participants to assess which clinical data should be seen as sensitive, and some highlighted the context-specific nature of sensitivity (see Table 4 for specific examples).

*I think all patient records tend to be very confidential. It doesn’t matter which part of it. It’s all sensitive. (P32 Malindi Researcher).*

*I think it’s very hard to narrow down the definition of what is sensitive data because it’s basically a cultural determination. I mean in our cultural context, HIV is much more sensitive than Hepatitis B, there’s no logic to that. (P30 Kilifi Senior researcher).*

**Table 4:** Complexities seen for patient autonomy in routine public health reporting

Example 1 Multiple drug-resistant TB cases: Where exact information on the patient’s location (residence) must be shared to allow adequate follow up. This type of reporting would be done without seeking the patient’s permission or necessarily informing them that the report had been made. Any infringement of rights to confidentiality or increased risks of stigma in this case were felt to be reasonable given the wider public health benefits as well as the individual’s own health risks. Example 2 Rare diseases: In routine reporting or publishing on cases of rare diseases, an issue raised by a hospital manager and senior researcher was that the much greater risk that a patient could be identified and linked to a particular health condition meant that patients should be made aware of and be asked for permission for this data to be used in audits. In this case, there was little public health benefit seen to outweigh the risks of individual infringement. *‘…in rare circumstances, rare infectious diseases or any other condition which is not as usual that’s where we sometimes tell the patient that expect this and this and this but in routine cases like malaria or URTI, it’s just routine.’ (Kilifi manager KLFM 11)*	

Given this uncertainty about what might constitute sensitive data in different contexts, some participants argued that individual engagement would provide a means of allowing patients to make their own assessment of the acceptability of re-use. Similarly, a few participants noted that some public engagement activities (including seeking input from community advisory boards) might be an important way of identifying which data should be considered sensitive in different contexts, and helping to identify any risks associated with sharing such data.

### Arguments against sharing information on clinical data re-use with patients or publics

At the same time as supporting information sharing with patient and a wider public on clinical data re-use, many participants raised a number of interrelated counter arguments that highlight potential unintended impacts on public health and resource burdens. These arguments revolved around the fear that information sharing around data re-use practices could raise concerns among patients and the public and have the unintended effect of patients refusing to share EHRs and therefore having important public health impacts. Arguments were also raised around if and what forms of consent would be required for the re-use of patients’ routine clinical data and about the resource burdens that these forms of information sharing imply.

#### Creating awareness may lead to refusals that undermine quality of care and public health

Given that participants strongly recognized the importance of some forms of clinical data re-use to support quality of health care and public health planning, many expressed concern that providing information to patients or the public on clinical data re-use could lead to patients refusing to allow data re-use. This risk was seen as serious, particularly by policy makers, with refusals from even a small percentage of patients having important implications for the value of the data. Patients were seen as particularly likely to refuse data re-use where concerns, misconceptions and rumors arose about the ways in which data might be used.

The emergence of such concerns and misconceptions was seen as likely to have further repercussions. Firstly, misconceptions and fears could lead individual patients to refuse to have particular routine medical tests done, or provide certain types of clinical information. Such refusals could adversely impact the care and outcomes for individual patients. Secondly, such fears could undermine trust in public health services that were seen to be implicated by worrisome practices, even potentially leading to the boycotting of certain health providers or facilities. These negative outcomes were associated with both individual patient information sharing and most forms of public engagement, and risks were seen as highest in the latter context.

Towards a similar end, a few providers and hospital managers argued that consent for data re-use had been implicitly given by patients who chose to seek care at public hospitals:

*By them coming in … [or] choosing to come to the laboratory for services … in one way or the other it’s like assumed you are offering this data, to be aggregated later (P33 Malindi healthcare manager).*

Similarly, a senior researcher noted that some forms of public engagement about clinical data re-use were already in use (such as display boards with aggregate patient information outside government Health Centres), so that the public should already be aware of this practice.

#### Extra resources needed to support good communication come at a cost

An additional argument against sharing information with patients on clinical data re-use concerned the practical difficulties and resources involved, especially in public hospitals often characterized by high numbers of patients and limited resources, including numbers of providers. This point was particularly emphasized by health providers and managers, arguing that time spent engaging patients in this way would undermine time spent in attending to patients in other more important ways, and risk impacting standards of care:

*‘What we need to appreciate is that most of the public hospitals, they are congested … and the health workers are very few. So if we say we are going to explain to our patients what we are going to do with their clinical data, it might take long and at the end of the day, very few patients will be served. Someone who came in the morning might end up being attended to in the evening’ (P06 Kilifi healthcare manager).*

The costs involved were seen as particularly significant given the unfamiliar and complex nature of the information to be shared, taking time and skills to communicate effectively. Participants were particularly concerned about challenges in communicating about areas seen as complex, including processes of de-identification, and uses for known or unknown future research. In this context, many participants expressed further concerns concerned about increased communication challenges in a setting where many people using public health facilities were likely to have had low levels of exposure to formal schooling. At the same time, while many recognized that communication could present an additional resource burden, others noted that such challenges were typical of medical practice in general and not in any way unique:

*‘ … communication is key … while I attend my patients I have to talk to them, I have to make them feel comfortable you know, so I just think it’s about attitude.’ (P03 Kilifi health provider).*

Finally, where information sharing on clinical data re-use *was accompanied by a requirement for patient consent*, an enduring ethical issue around the validity of consent in contexts of limited health care access was raised by a several participants in Malindi [34]. In this context, seeking consent for data re-use was seen as potentially meaningless, since patients might often feel that their treatment would be contingent on, or importantly influenced by, this agreement:

*‘So, when I came here, I needed to be cured and you’re telling me that I’m going to treat you but be aware that one of the one of these days your data may be used for an evaluation. Of course, I’m forced to say yes, because it’s like if I don’t say yes, I won’t get the treatment. So, for me still ethically it’s not right.’ (P33 Malindi healthcare manager).*

### Considering scenarios: Arguing for and against information sharing

In practice, the main arguments for and against sharing information on clinical data re-use practices outlined in the previous section, and summarized in Table 3, were drawn upon in a very dynamic way during discussions around each scenario. In this section, we illustrate how these arguments were weighed up against each other for each scenario in turn, including for routine monthly reporting; for evaluation activities; and for comparative effectiveness trials (non-randomised and randomised).

#### Scenario 1: Re-use of clinical data for routine monthly clinical data reporting

Given the public health importance of this form of clinical data re-use, and perceptions of a risk that some patients might refuse to allow their data to be used in this way, the arguments against individual informed consent for this form of clinical data re-use were strong and prominent, but public and patient notification were often seen as potentially beneficial, as described in the following paragraphs.

The strongest version of this argument was that under no circumstances should an important public health good in re-using EHRs be compromised by the need to promote patient autonomy. This argument was strengthened by an understanding that the data would normally be shared in aggregate form (which was considered minimally sensitive given low risk of individual re-identification), and would be used primarily for public health gain by the Ministry of Health.

*When making a count we are not using the names, so the identity goes away completely … if you are going to say that we are using [name mentioned]… then that becomes an issue (P25 Malindi healthcare manager).*

For some, on this basis, individual consent to re-use was seen as unnecessary and too risky. In this context, the counter-arguments in support of engagement (for example, concerning rights to know, the value of openness, accountability and normalizing data re-use) were felt to be relatively unimportant, and the costs of creating awareness were seen as an unnecessary burden to public health resources.

*Sometimes even you as an individual your rights sort of ends where there is a bigger purpose at hand (P32 Malindi researcher).*

In contrast, a second and more common type of argument drew strongly on the ideas described in section A (arguments for information sharing) around the value of openness and showing ‘nothing to hide’. Most individuals felt that that patient and public notification, as opposed to informed consent, would not present a risk to an important public health function, but would instead promote an important set of supportive values:

*If we don’t inform them then they wouldn’t … feel like they are part and parcel of what you are coming up with … [that] they have contributed in one way or another (P09 Kilifi healthcare manager).*

*They should be informed so that they can get the clear picture of what it is happening in the hospitals, and how that information is important to the [National and County] government (P31 Malindi researcher).*

Overall, there was more support for public than patient notification, mainly on the basis of the resource costs for the latter. However, showing the difficulty some participants experienced in making a judgment about these issues, many felt that no simple answer was possible:

*And then probably a more important thing would be if they do not consent, its fine for one or two, what if there’s mass refusal to consent and yet this is national data? I don’t know how to answer you whether it’s a yes or no. It’s a very difficult thing to try and answer (P32 Malindi researcher).*

Although monthly reporting was seen as the least sensitive form of clinical data re-use, the perceived sensitivity and level of data aggregation in audits were raised as important potential influences on the need for patient engagement. Two examples were raised around forms of routinely reported data that may be very sensitive and have a high risk of de-identification, with differing perceived implications for patient engagement, shown in Box B.

#### Scenario 2: Re-use of clinical data for evaluation activities

Shifting the scenario from considering routine audits to forms of clinical service or guideline evaluation was often a very subtle move; many participants were initially unable to see clear differences. However the main change in attitudes linked to this change of scenario focused on the identity of the main end-user, as a way of building confidence in the main purpose of data re-use and the patient protections likely to be in place. The key distinction was data re-use by the Ministry of Health (assumed to be solely for direct support to public health services) or non-Ministry of Health end users (where the purpose was seen as less clear), as described below.

##### Evaluations run by the Ministry of Health to improve services in public hospitals

Given confidence that data would be re-used in ways that would positively impact public health services, a majority of participants felt that patients should be aware that their data are being used for Ministry of Health-led evaluations, but that it was not necessary to seek consent for this use, as commonly described for audits. Hospital managers and senior researchers were particularly clear that the use of patients’ clinical data by the Ministry of Health for both audits and evaluations is an important and legitimate use of the data that falls within their remit to provide good quality public healthcare. Some participants also pointed to the fact that hospital board members had a role to represent the community and act in the interest of patients.

Arguments for patient notification, as opposed to consent, were made more strongly than for the audit scenario, particularly on the basis that individuals have a right to know how their data are used and that explaining this use demonstrates respect to patients. Similarly to the audit scenario, there was general support for public notification which – as shown in section A – was sometimes felt to already be in place in peripheral health facilities to some degree, through the public display of information on numbers of cases seen across different disease categories.

##### Evaluations run by non-Ministry of Health stakeholders

This group of evaluations included those run by non-governmental organizations, masters’ students from national universities or researchers from international collaborative institutions. In this situation it seemed to be less clear what the main purpose of the evaluation would be, and participants were more likely to expect that consent would be sought from patients. This shift in attitude was underpinned by several concerns. One argument was that if patients’ understanding is that their data will be used for their own care, and this is tacitly extended to support public health services they personally access (as for Ministry of Health-led audits and evaluations), it would not be reasonable to extend this permission to other organizations. Particularly for individual-level data, re-use by non-MoH organisations was seen as sensitive:

*Where someone ... who is not a medical staff involved in patient care is going to open up your patient record and start ferreting through for particular details they want... if they’re from outside the MoH, I don’t think that should be allowed in the absence of informed consent (P30 Kilifi researcher).*

A wider issue of trust is hinted at in the above quote, and was referenced in different ways across these discussions, including a comment for example that ‘*many NGOs have their own personal interest which they don’t share … ’ (P34 Malindi healthcare manager).*

In contrast, a small group of participants were concerned that information sharing about non-Ministry of Health evaluation activities would lead to an increased risk of rumors and subsequent undermining of public trust, as has been described earlier. For this smaller group, neither informed consent, or individual or public notification were felt to be advisable.

Participants also discussed that evaluation activities using hospital clinical records are often conducted retrospectively rather than prospectively, removing the possibility of specific prior informed consent for data re-use. However, most participants felt that if patients were still in the hospital, it would be good practice to create awareness about potential future data re-use in this way. In addition, on prompting by the research team, a number of respondents felt that seeking broad consent for future uses of clinical data alongside appropriate forms of governance might also offer a potential solution, as discussed further in section D.

#### Scenarios 3 & 4: Re-use of clinical data for pragmatic clinical trials: Non-randomized and randomized trials

In this section, we describe views on information sharing and governance in relation to forms of pragmatic clinical trials (PCTs), including non-randomised (non-RPCT) and randomized (RPCT) trials. In doing this, we show a turn to a more regulatory stance, including the greater prominence of individual informed consent requirements, particularly for RPCTs. At the same time, researchers in particular noted that ‘evaluation’ and ‘research’ may often be very closely related activities, illustratively described by one as ‘nose-mouth close’ *(P24 Kilifi researcher).*

While the processes involved in non-RPCTs and RPCTs were always carefully discussed in each interview, participants almost always interpreted this form of LHS as ‘research’. One underlying reason was that their technical nature led participants to feel that PCTs would not be run by Ministry of Health actors alone, but by research teams potentially in collaboration with the Ministry of Health. Given a view of PCTs as a form of research and the involvement of non- Ministry of Health stakeholders, many participants viewed informed consent as an automatic requirement, for reasons that were seen as both intrinsically and instrumentally important, as discussed below. At the same time, an important influence on views was awareness that non-RPCTs (like evaluations) may be conducted retrospectively, making patient consent more practically challenging, while RPCTs would always be a prospective activity.

##### Seeing informed consent in PCTs as intrinsically important:

The majority view overall was that PCTs were so close or equivalent to ‘research’ that patients should generally be asked for informed consent, to respect their autonomy to make a choice about involvement:

*I think in as much as we are using standard drugs that have already been … have been approved but this is... there is a research element there, so consent should be sought (P02 Kilifi health provider).*

These views were particularly pronounced where randomization was involved but were also held by some for non-RPCTs. In this way, a senior researcher considered that randomization processes would inevitably be associated with a shift in the doctor-patient relationship. While patients typically assume that their doctor will make a decision about their treatment based on an assessment of individual needs, in an RPCT the doctor will make this choice using a research process. It in this situation, the researcher argued that patients have the right to know that this change in the doctor-patient relationship has occurred:

*So, most of the time as a doctor when you come and see me … what I’m putting down is that I’m confident that this is the thing [prescription] that’s going to make you better and I’m sort of owning that responsibility. In this case [RCPT], we are making the decision together [through consent]… that we don’t know [which drug works best]… we are starting from the point that this is not what we ordinarily would think.’ (P29 Kilifi Senior researcher).*

A similar ‘right to know’ argument was based on concerns that data re-use in PCTs had clearer and more unique benefits to non-Ministry of Health stakeholders than public health services, increasing the need for patients to be aware of this use. Concerns were also raised about the potential harms of involving patients in a RPCT, where the large sample sizes involved would mean that rare side effects of licensed treatments might be uncovered in a RPCT. It was felt that this possibility underlined the importance of choice and ought to be explained to participants.

At the same time, particularly for non-RPCTS, a few participants (a provider and two researchers) argued that clinical data could be re-used without patient consent, given that there is no interference with routine care, and that any refusals risked undermining the important public health value of the activity. While this perspective was not universally discussed, where participants recognized that non-RPCTs may be conducted retrospectively, this was also seen as practically disbarring the possibility of prior individual informed consent.

Even for RPCTs, a minority of participants argued that patient awareness but not consent was important, given that these activities involved the use of approved drugs with no additional burdens on patients, were conducted within routine clinical setting in which patients had already given implicit consent for their providers to make choices on their behalf, and given concerns that signed consent could generate rumours that drugs were being tested on patients.

##### Seeing informed consent in PCTs as instrumentally important:

Participants raised a range of arguments in support of seeking informed consent in PCTs that were linked to positive outcomes of giving, and negative outcomes of withholding, information. One underlying position was that PCTs were more ‘active’ than evaluations or audits, involved more planned and specific uses of individual patient data and higher risks to confidentiality (given that potential data users were not involved in clinical care and the potential for wider dissemination of findings).

As a negative outcome of failing to share information, some argued that a failure to respect the rights of patients to understand how their EHR were being used could, as well as being intrinsically wrong, lead to litigation:

*They’re [PCTs] cases of rights, advocacy for patients’ rights and even some clients may threaten to sue you if you did something without their consent (P26 Malindi health provider).*

Others saw that the inclusion of individual patients in an RPCT without their understanding could prevent patients from accessing drugs of their choice that were personally ‘known’ to be more effective, leading to less good clinical outcomes. While this position seems to contradict assumptions of clinical equipoise for PCTs, placebo effects could not be discounted. A potentially more robust view was that failing to share information could lead to a loss of trust in health systems, if later discovered (as outlined in section A). A particular version of this concern for RPCTs (and to a lesser extent non-RPCTs) was the need to avoid creating public concern around a lack of medical understanding about which drugs ‘work best’, leading to facilities implicated by this uncertainty being boycotted.

As a positive outcome of sharing information, better understanding was linked with strengthening compliance with randomization and drug use, and hence the validity of a study. Looking to the longer term, a senior researcher highlighted the historical context of research ethics for clinical data re-use, recognizing that strategies like informed consent can be seen as a response to wider societal norms and concerns about research and researchers. This participant commented on the long term role of consent and public engagement in building confidence in public health research uses of routine clinical data, a prominent value described in section A, so that in future there may be less need for individual informed consent for data re-use:

*And if you get to a situation where just everybody who’s admitted to hospital is expected to be in randomized clinical trials, then you can shift the culture (P30 Kilifi Senior researcher).*

Forms of public engagement, including general notification, around data re-use for evaluation and CER purposes were seen as challenging to develop, but critical to tackle since families at home will always have some awareness of what is happening to their relatives and neighbours in hospital. It was also argued that providing relevant public information could save time in explaining PCTs at the hospital. This position was supported by a view that patients and families would value the public health potential of PCTs, particularly non-RPCTs, if they understood what was involved, given the negligible patient burdens.

### An emerging value of Ministry of Health governance for clinical data re-use

Given concerns about the ways in which clinical data might be re-used, particularly by non-MoH actors, a key issue agreed upon by participants was that all such uses, whether or not patient consent was sought, should be approved by and generally be undertaken in partnership with the MoH. Effective governance was seen as particularly important for any retrospective clinical data re-use activities where prior informed consent would not be possible, including some evaluation and non-PCT activities. While, given time limitations, the topic was discussed in only a few interviews, oversight was seen as particularly important for forms of individual prior broad consent were proposed, that is, seeking patients agreement to undefined future uses of clinical data for evaluation and research purposes [35].

Effective governance was particularly emphasised for PCTs, since these were generally seen as more like traditional research activities, and more often led by non-Ministry of Health organisations. Governance was also seen as important for PCTs (particularly RPCTs) to support researchers and institutions in case policy-relevant evidence was later challenged:

*Okay fine you say the drugs [used in a PCT] are all licensed, but the fact that if you find that maybe drug X is no longer valuable it can have an impact economically to the company... and people could even later come and sue the company (P29 Kilifi Senior researcher).*

For evaluation activities undertaken by NGOs in partnership with the MoH, existing MoH governance processes were broadly seen as sufficient, in checking that the purpose of the data use was reasonable, adequate measures had been put in place to protect patients and that the evaluation/research was relevant to the local population/context. The presence of community representatives on hospital boards was reassuring in this respect.

For PCTs, where research institutions were thought more likely to lead the activity, participants noted that oversight would often be through institutional Research Ethics Committees. A recommendation from senior research and MoH managers who raised this topic was for increased linkages between research and MoH governance processes to promote efficiency and more effective oversight of these collaborative activities.

## Discussion

Against a background of proposed opportunity to fast-track healthcare system improvements in LMIC facilities with effective electronic health records, our study aimed to assess the views of health providers, managers and researchers in coastal Kenya on the acceptability of re-using anonymized individual patient data for quality improvement and comparative effectiveness research, including the role of informed consent and individual and general notification processes. We believe that these findings, while limited to the views of participants with expertise in health research and care provision or management, provide insights into likely values around core ethical issues of consent and governance for LHS approaches in an LMIC setting. Our findings may also have value in planning critically important research to explore patient and public views in such contexts.

The overall nature of many of our findings concur with those reported from HIC settings including the USA, UK, Canada and Australia [22, 27, 29]. Firstly, the views of participants on information sharing processes were often very varied, and were related to aspects of the context of clinical data re-use being described. In our study, diversity of views has at least in part arisen from a detailed consideration of different aspects of clinical data re-use, including the type of data, the purpose of re-use and the main users. The detailed nature of our findings has allowed us to develop a nuanced understanding of determinants likely to underpin attitudes, and provides insight into challenges that may be specific to this and other similar contexts, as we go on to discuss in the following paragraphs.

Relatedly, across our discussions nearly all participants experienced difficulty in distinguishing between the fundamental nature of activities in the different scenarios discussed (audit, evaluation and non-RCPTs and RPCTs), highlighting ‘greyness’ in these distinctions, as described in the background to this paper. This continuum between quality improvement and research activities has also been described in the literature as presenting challenges for research ethics committees, in identifying which activities require ethics review and patient consent and which ones do not, and risking over-protection of patients in quality improvement research or under-protection in pragmatic forms of research [6, 36].

A second point of connection between our study and the literature is that a well-recognized fundamental ethical tension for information sharing on clinical data re-use emerged strongly in our data; that is, of the need to balance patients’ rights to know about and control clinical data re-use with the public health good served by these processes [37]. While both sets of values were almost universally seen as important, in common with other studies, our participants were mixed in their views about how they should be balanced in specific situations, with contextual influences again having an important role in explaining diversity [38].

Across the following sections, we aim to discuss the contribution our data makes to clarifying how values of a public health good and patients’ rights to know can be balanced in this setting; highlight the strongly emerging issue of trust in relation to the role of a national Ministry of Health in clinical data re-use processes; and underline the importance of governance structures given the complexity and context-specificity of many of the ethical and practical issues involved in re-using clinical data.

### Balancing a public health good and patient’s rights to know or control clinical data re-use

Both public health benefits and patients’ rights to information on clinical data re-use were supported as important values across this consultation.

*Linked to public health benefits*, an important consideration was the extent to which initiatives were led by the Ministry of Health. Where initiatives were led by the Ministry of Health (mainly scenarios 1 and 2), data re-use was seen as likely to feed directly into areas of important and locally relevant healthcare planning, and therefore have high public health value.

*Linked to patients’ rights to information,* these were particularly stressed when the data re-use activities were thought to have characteristics of research, which in turn seemed to evoke a need for regulatory frameworks (mainly scenarios 3 and 4). Patient rights to information were also more prominently claimed where non-Ministry of Health partners were involved. In this situation, the need for patient awareness and/or consent was linked to a perception that public health benefits were less clearly and immediately anticipated, higher risks of de-identification and associated harms were likely, and greater gains seemed likely for ‘external’ versus Ministry of Health partners. In practice, the involvement of non-Ministry of Health partners, such as research institutions, and data re-use activities having more research-like features are highly likely to occur together. A limitation of this analysis is that well planned research should of course aim to have important public health benefits, but any such gains are likely to be in the longer term, more widely applicable and not necessarily easily translated into policy in the contexts where clinical data are accessed.

**Where public health benefits of clinical data re-use were felt to be particularly likely and important**, differences in opinion around the value of information sharing often turned on the anticipated outcomes of the engagement processes involved. These conflicting views, described in the findings section, were that individual and general information sharing would either i) generate concerns, undermine trust and lead to refusals, or, conversely ii) generate support and build trust.

An immediate implication of this finding is the need for more empirical research to explore the likely outcomes of individual and public information-sharing on clinical data re-use, before future policies on individual or public information sharing are developed. Patient and public perspectives would be a critical component of such research. The nature of data was an important influence in this balance, and would need to be taken into account in future research. Where data were seen as routine, non-sensitive (itself a subjective judgement) and with little risk of re-identification, information sharing was seen as less important, and therefore not worth risking potentially important adverse outcomes. Conversely, for more sensitive data with greater risks or re-identification, patient awareness and/or consent for data re-use was a more prominent concern.

However, given the resource costs of including individual informed consent processes for clinical data re-use, and recognising that the views of patients and publics would be a critical consideration in developing such proposals, our findings suggest that effective individual or general notification processes may be an acceptable alternative for data re-use in this setting, as long as anticipated public health benefits are strong, local and near term, and accountable governance mechanisms exist. [26, 39]. This strategy places emphasis on a valued public good in clinical data re-use, while limiting risks to individual autonomy, public health resources and public trust.

**Where clinical data re-use was seen as ‘research-like’ and non-Ministry of Health partners were involved**, participants were almost universally more likely to require the use of an individual informed consent or notification process. This finding accords with others in the literature, suggesting that patients would prefer to be asked for permission before their data is used in PCTs [22, 40]. In particular, in our study, where learning initiatives involved prospective random allocation of patients to different approved treatments (scenario 4), there was a universal view that patient consent would be needed.

In scenarios 3 and 4, participants identified a range of reasons that patients should be aware and often give consent for clinical data re-use. In keeping with other studies [27, 29] participants particularly noted issues of trust in the doctor-patient relationship. In this way, patients should be aware that their doctors’ motivations in choosing treatments in a RPCT are different to those normally used, and are not based on clinical judgment of a patient’s best interests. Also supporting the literature, we noted arguments for awareness about participation in a RPCT related to perceptions of differences in the levels of risk involved, reflecting challenges described for IRBs in assessing risks in CER [41].

A further practical issue noted in our study for a RPCT was the need for patient consent to be part of a system of research governance underpinning good clinical practice, particularly in situations where research outcomes may have direct financial and legal implications for the public health sector and industry partners. Since the concept of a pragmatic trial is a variable one, moving from ‘most pragmatic’ at one end to ‘most exploratory’ at the other, as described in the PRECIS-2 model [11, 12], this point suggests that the intended policy implications of a learning initiative may have a bearing on the choice of research design. As research designs move from more to less pragmatic, more formal processes of patient consent may be required.

A final point on information sharing for PCTs is that it was clear that the use of the term ‘pragmatic clinical trial’ during our interviews may have generated an immediate assumption that the activity being discussed was a classical form of research, invoking a need for individual consent and other considerations of autonomy. In practice, as part of the pragmatic trial continuum described above, some PCTs may act more as evaluations, for example, where a retrospective comparison of the effectiveness of existing health interventions is based on the use of anonymized patient clinical data. There may therefore be a need to re-evaluate the language used in communicating about atypical forms of ‘research’ in future consultation activities and potentially in communication strategies.

### Emerging issues of trust

Overall, the issue of trust emerges as key across our findings, in different ways. Firstly, as described here and from other settings, trust in the doctor-patient relationship suggests a responsibility for doctors to make sure that their patients are actively aware and engaged in learning healthcare activities [17, 22]. Secondly, loss of public trust in health systems was described as an important unintended consequence of engagement in this study, as has occurred in the UK in similar circumstances [42]. However, as noted earlier, the relationship between trust and communication may not be a straightforward one, with communication about learning healthcare activities being seen as both risking loss of trust and as potentially being trust-building. Loss of trust was seen as potentially occurring through patient fears about their data being used for commercial gains, identifiable data being shared with third parties and concerns over the efficacy of drugs used in randomized pragmatic clinical trials. The loss of trust in doctors or the health system can result in patients engaging in ‘privacy protective’ behaviors, including boycotting hospitals where data is used for learning purposes or withholding sensitive information or refusing routine medical tests, inadvertently impacting on the quality of care that can be provided [26, 43, 44]. Clearly, these forms of loss of trust can have further implications for expectations of a public health good from LHS activities.

Issues of trust were most marked when non-MoH partners were involved in learning activities, linked to concerns about fairness and transparency in identifying who the main intended beneficiaries of such research activities are. We and others have reported similar public concerns about fairness in the distribution of burdens and benefits in related practices of re-using public health research data, concluding that core elements of an ethical data sharing model should assess likely scientific progress, minimize risks of harm, promote fairness and reciprocity, and build and sustain trust [45]. In this way, and in common with others [13, 37], our findings emphasize the importance of recognizing that LHS activities must be grounded in active collaborations between researchers, health managers and providers, patients and the wider public that promote trust and can identify context specific elements of LHS activities that serve this function.

### The importance of oversight and governance

Given the many influences that were seen to change the way that ethical issues of public health benefit and patient rights should be balanced, many of our participants emphasized the importance of strong governance systems to provide context specific oversight of the science and ethics of LHS activities. This importance of governance is recognized in the literature, alongside a need for greater harmonization of the different bodies that may be involved [2, 46]. Similarly, senior managers in our study pointed out that, while different LHS activities seem to follow a continuum rather than falling into discrete groups of activities, learning initiatives classed as quality improvement are likely to be submitted by NGOs for approval by institutional review bodies in hospitals, while those classed as LHS activities, particularly pragmatic trials, are generally developed with technical support by research teams and will generally be reviewed by ERCs/IRBs.

From the perspective of health managers, providers and researchers in our study, the current systems of Ministry of Health governance seem likely to be appropriate for routine clinical audits to support health service functioning. This form of oversight could act as a proxy for individual consent processes, but would be usefully supported by carefully developed patient and public notification systems. In contrast, the separate forms of governance currently in place for other learning initiatives, such as QI (Ministry of Health governance) and PCTs (research ethics governance) are in need of greater harmonization, which could promote effectiveness and efficiency given the different strengths and challenges of these governance mechanisms.

Across the literature, the challenges for ERC/IRBs in assessing different forms of LHS are well recognized and linked to bureaucratic delays that frustrate the underlying goal of continuous learning [6, 15, 18, 25]. Faden et al. (2015) have proposed a specific ethical framework for LHS review that adds new obligations to ‘avoid imposing nonclinical risks and burdens on patients, reduce health inequalities among populations, conduct responsible activities that foster learning from clinical care and clinical information and contributes to the common purpose of improving the quality and value of clinical care and health care systems’ [20]. Highlighting ongoing controversy in this area, the framework has been critiqued as potentially under-representing patient interests in some instances [47]. Perhaps most saliently, recommendations on the ethics of LHS also recognize that the types of consent and governance processes needed will depend on the ‘maturity’ of the LHS and the wider health system in which it is embedded [20], where a mature LHS has been associated with established norms for learning activities and regular information sharing with patients about learning activities [7, 25].

In our situation in Kenya, the notion of a mature LHS is still remote. More research is clearly needed to explore public and patient views on the acceptability of re-use of patient data for different purposes in this and other LMICs settings before clearer recommendations can be considered. As before, more research is also needed to learn how to communicate effectively with patients and the public in our setting about different forms of learning activity that may take place within hospitals. At present, we would recommend closer communication between research and healthcare governance activities within hospital settings to strengthen these processes overall. For now, where researchers lead on learning healthcare activities, review by ERC/IRBs will continue to be needed and where feasible (for example, for prospective studies) consent from patients sought. Over time, and with greater public and patient awareness and support, learning healthcare activities may become normalized to some degree. Such a mature learning healthcare model would continue to require locally informed governance systems that can appropriately assess the range of learning activities that might be proposed, and identify situations in which patient consent remains an ethical requirement.

## Conclusions

Based on the views of health managers, providers and researchers in a rural Kenya context, this study shows the complexity of assessing the role of individual and public information sharing for hospital-based learning healthcare activities, given multiple forms of diversity around the nature of the LHS process itself. The findings suggest that while individual and public information sharing on LHS are important, planning these activities should take account of any potential to undermine capacity to inform important public health activities or public trust in healthcare systems. Two dimensions to LHS are central in assessing the type of information sharing needed; which organization is running activities, and who will primarily benefit from the activity. For LHS activities run by the Ministry of Health to support audit and quality improvement in public services, individual consent may be less necessary, while individual and public notification are important but in need of careful development. For LHS activities run by other stakeholders, including evaluations and PCTs, both individual notification or consent processes and partnerships with the Ministry of Health are likely to be key. Given challenges around individual consent and sometimes notification for LHS, governance mechanisms are particularly important. Greater collaboration between existing and currently separate Ministry of Health and Research Governance systems would strengthen and may expedite processes towards the development of more mature and locally responsive learning healthcare systems.

## Additional file

Additional file 1: Focus Group Discussion and Interview tools (DOCX 46 kb)

## Abbreviations

CER: Comparative Effectiveness Research
EHR: Electronic Health Records
ERCs: Ethics Review Committees
HIC: High Income Countries
IRBs: Institutional Review Boards
KCH: Kilifi County Hospital
KWTRP: KEMRI Wellcome Trust Research Programme
LHS: Learning Health System
LMICs: Low to Middle Income Countries
MoH: Ministry of Health, Kenya
MSCH: Malindi Sub-County Hospital
NGOs: Non-Governmental Organizations
NHS: National Health Service
PCT: Pragmatic Clinical Trials
PRECIS: Pragmatic Explanatory Continuum Indicator Summary
QI: Quality Improvement
QIR: Quality Improvement Research
RPCT: Randomized Pragmatic Clinical Trials
UK: United Kingdom
USA: United States of America
