# Supplementary material for: Kenyan health stakeholder views on individual consent, general notification and governance processes for the re-use of hospital inpatient data to support learning on healthcare systems
Source: BMC Med Ethics. 2019 Jan 8;20:3. doi: 10.1186/s12910-018-0343-9 (PMC6325859; doi:10.1186/s12910-018-0343-9)
Supplement: Supplementary file 1 — Focus Group Discussion and Interview tools (DOCX 46 kb) [file 12910_2018_343_MOESM1_ESM.docx]

**Supplementary file 1: Interview guides for Clinical Learning Systems Project**

The following In-depth Interview Guides are included in this section:

- IDI guide for Hospital Providers
- IDI guide for Researchers
- IDI guide for research field workers (front line)
- IDI guide for Hospital Managers

## Clinical Learning Systems Project: IDI guide for Hospital Staff (Providers)

*Introduction: In this discussion, I would like to understand your experiences and views around ways that routine clinical data are (or could be) used to promote public health in addition to for individual patient care. At present, these data are already used for routine monitoring e.g. in monthly reporting statistics. There are other potential uses for clinical data – with patients’ names taken off and either used individually or aggregated. I would like to get your views on the use of data in these ways.*

1. **Type of data & their management/storage**: Confirm information in table on types of clinical & laboratory data and ways these are managed, stored/archived over time. Make any additions/changes.
2. **Routine monthly reporting:** We know that clinical data are routinely collated and reported from County to National MoH, to inform mapping of burdens of disease and plan budgeting and services across the country.

- To what extent do you think patients are aware that ‘their’ clinical data - without names or other identifiers - are used in these ways?
- Do you think they should be aware? Why do you think this is/is not important?
- If so, how should this be done? [*Probe: including should give information or ask permission?]* Why do you think this? Any experiences of this being done, and how did it work out?
- Do you think that the public should know that patient data is used in this way? Why do you think this is important?
- If so, how should this be done?

1. **Use for audit or evaluation**
2. Are clinical or laboratory data currently used for auditing or evaluating clinical services in this hospital? *[By audits we mean a process that seeks to improve patients care and outcomes through systematic review of care against set standards. Service evaluations on the other hand involves assessing how well a service is achieving its intended aims.]* If so, can you tell me about this?

*For a typical example of a known audit or evaluation process:*

- Which data were used, over what period of time and what analyses done?
- How often does this happen?
- Who was conducting the audits/ evaluating processes? Whose responsibility is this? [*Ask for access to any auditing or evaluation reports, if possible*]

*If no known example*, discuss the following situation:

| **Scenario A: An MoH-led evaluation**  A public health manager in the County Health Team uses clinical and laboratory data from individual patients (with names taken off) **to evaluate whether new guidelines** that have been introduced in different hospitals for clinical care are working well (i.e. improving clinical outcomes). |
| --- |

1. For the ‘real life’ example above or for scenario A or B:

- In this situation, do you see any differences in whether patients are aware compared to the monthly routine reporting?
- Do you think any differently about whether patients should be aware? If it’s different, why do you think this is important in this situation but not the previous one?

*[****Probe:*** *When you think about this going forward, should the hospital begin* ***informing*** *patients about this use of clinical data NOW?* ***Why?*** ***How*** *can this be done? What about asking patients for* ***permission*** *NOW going forward? What kind of permission?* ***Why*** *do you think this is important?]*

- What about information to the public – do you feel the same or differently about this for audits/evaluations as for monthly reporting? Why? And how should this be done? *[Probe: Giving information only or also asking for permission]* Any experience of this being done, and how did it work out?

1. **MoH/NGO partnerships for national & international use**:

In the previous scenario, we have talked about the Ministry of Health using clinical data for monthly reporting or to evaluate new guidelines. we would now like to consider the situation where it’s NOT the ministry using the clinical data but an NGOs, which may be working in partnership with MoH on provision & evaluation of services, at national and international levels.

| **Scenario B1: An evaluation conducted by an NGO in partnership with MoH**  An **NGO** is using clinical information in an aggregated form (without names) collected across a number of different hospitals in Kenya to evaluate the quality of malaria case management, with the aim of improving services in **all hospitals** in Kenya and other similar settings. |
| --- |

- Do you think patients should be aware their clinical data are being used in this situation? Why do you think this is important? [Check for difference in opinion and if so; why do you think this is important in this situation but not the previous one?]

*[****Probe:*** *When you think about this going forward, should the hospital begin* ***informing*** *patients about this use of clinical data NOW?* ***Why?*** ***How*** *can this be done? What about asking patients for* ***permission*** *NOW going forward? What kind of permission?* ***Why*** *do you think this is important?]*

- How should this be done? [Probe: *Should give information or ask permission? Any experiences of this being done, and how did it work out?*]

ALSO:

- In situations where getting permission from patients is not really possible, what should be done?
- We talked earlier about situations in which it might be important to give information about the uses of clinical data in a hospital to the ‘public’ who use that hospital. Do you think this (scenario B) is one of those situations? Why do you think this is important here?
- If so, how should this be done? Any experiences of public information/engagement, and how did it work out? *[Probe for general information e.g. posters or individual explanations and/or consent processes]*
- Would this also apply to situation in Q3? *[remind about scenario A]*

1. **Use of sensitive data**: For situations where clinical data might be seen as **sensitive**, does this affect your views so far on whether and how patients or the public should know about the ways clinical data might be used (beyond individual patient care)?

What kind of data would you include as **sensitive**? [Probe: Should they be handled differently?]

**Scenario B2: Evaluation using sensitive data**

Donors for HIV/AIDS programmes that support service delivery in specialized government outpatient clinics use clinical and laboratory data collected from clinic users (with names and other identifiers taken off) to support evaluation of the programme they fund.

- In this situation, do you think patients should be aware? Why? Do you see any differences in what information patients should be given or what permissions asked for? What do you think should be done differently, and why do you think this is important in this situation but not in others?
- What about information to the public – do you feel the same or differently about this for sensitive clinical information? Why and what should be done?

1. **Using clinical data for pragmatic clinical trials – No randomisation**

I would now like to ask you some similar questions about how researchers should provide information and/or seek permissions from patients for the use of their clinical data, but now in relation to a type of **research**. [This type of research is called a pragmatic clinical trial]. An example of this is given below:

| **Scenario C: Non randomized pragmatic clinical trial**  It is common in medical practice that doctors have several different treatments they can use for the same condition, and for it not to be known whether one treatment really works better than another. For example, many different antibiotics are recommended to treat particular infections, like boils, ear infections or lung infections.  In this situation, doctors tend to choose the treatment based on their own or their patients’ personal experiences/preferences. If there was more evidence about which treatments work best and in which situations, both patients and doctors would benefit. One way for researchers to do this is to compare routine clinical data on patient outcomes (e.g. how quickly or completely they got better after being treated by one drug compared to another). In this kind of research, the researchers DON’T introduce anything different to the normal practice. They just analyze the clinical data of patients who were treated to compare the effectiveness of different antibiotics used to treat the infection. |
| --- |

1. **In this situation**: *[Across all section below, include probes on any practical experiences in relation to information sharing or seeking permissions]*

- Does your views about whether patients should be given information about this use of ‘their’ clinical data change compared to the previous situations - where clinical data was being used for audits or when sensitive data was being used for evaluations of services? Should this include asking patients to give permission for this use?
- If different, why do you think it’s different in this situation and not the other? *[Probe: Should give information or ask for permission? Why do you think this is important?]*
- How should hospital management/ researchers make sure they know about /or ask them to give permission for the use of this clinical data? *[Probe for general information e.g. posters or individual explanations and/or consent processes – verbal or written]*
- *Again. where this is different to views expressed around scenarios A to D, probe for reasons behind these differences*

*[****Probe:*** *When you think about this going forward, should the hospital begin* ***informing*** *patients about this use of clinical data NOW?* ***Why?*** ***How*** *can this be done? What about asking patients for* ***permission*** *NOW going forward? What kind of permission?* ***Why*** *do you think this is important?]*

**Further probes**: Earlier we talked about whether some changes in the surrounding situation would change your views on whether and how information was shared with individual patients or the wider public. Can we talk about these areas again for this type of research (comparing the effectiveness of different approved treatments)?

- What if that data being collected was about HIV/AIDS? (compare with situation above)
- What if the research was being done by a researcher outside Kilifi:
- A master’s student from a Kenyan university as part of their course requirement?
- A senior researcher from another Kenyan research institute?
- A researcher working in another part of the world including another African country, and outside Africa e.g. Canada?
- What if the findings of the researcher’s work will be published in a science journal that could easily be read by many people?

1. **Using clinical data for pragmatic clinical trials - involving randomization**

The situation we talked about earlier (scenario C) - where researchers assess which of two approved treatments seem to work better by comparing patients - may not always give very good answers. The reason for this is that patients themselves vary in many different ways and that might also affect how well or quickly they recover from an illness.

I would now like to introduce a slightly different scenario to the one above. In this case, researchers are **NOT** using existing clinical data to determine the effectiveness of the two approved antibiotics. Rather, the research involves using a deliberate process to decide which of the two drugs each patient should be given. This process is called **randomization**. Randomization means choosing something at random, using a system based on chance (e.g. Tossing a coin, and making a choice based on which side the coin landed). Using a random process to choose which of the two treatments a patient is given, and including large numbers of people in the study, will make sure that any individual differences between the people in the study will not affect the final assessment. [*Use additional explanations for randomization and why its important where needed*]

**Scenario D: Randomized pragmatic clinical trial**

There are many different antibiotics currently approved and used routinely for treating pneumonia. For some of these antibiotics, it’s not known if they work better than others available. For example, let’s think about two such treatments, and call them antibiotic X and antibiotic Y. Both are already approved drugs and are in use at the moment. They are given in similar ways and have similar types and risks of any side effects or more serious reactions. (Serious reactions are very rare). It is therefore unlikely that patients or physicians would have a personal preference for one drug over the other. To find out if there are any differences between these treatments, researchers can ask physicians to prescribe one of these drugs based on a system of chance, and observe over time how well patients respond to the treatments. Over time, the outcomes of patients being treated with one of these two antibiotics can be compared to learn which treatment works best. Once this is known, all the patients can be given the option to change to that treatment.

- In this situation, what should patients who are being given one treatment or the other be told?
  - Should they be informed about the trial and given a choice about whether to be included, or only be treated as their physician would normally practice, as would be usual in research? Can you explain why you think this is important?
  - *[Remind about written informed consent processes] If think should ask for permission:* Should researchers use verbal or written consent processes in this situation? Why/why not?
- What if the research involved additional procedures to those that patients would normally experience? e.g. Filling a questionnaire? More hospital visits for blood sample collection? What difference would this make to your views on information giving to patients and seeking permissions for their involvement?
- What if the trial has been designed so that the physician has the option of using another treatment, other than the one randomly assigned, if they believe doing so is in the best interest of the patient?
- Should the wider group of hospital users/the public be informed that this kind of research activity is taking place in the hospital? Why do you think this? *[Probe for differences with earlier scenarios]* Do you see any difference in how hospital managers should try to inform hospital users in this situation compared to the other situations?

## Clinical Learning Systems Project: Draft Interview guide for Researchers

1. **BACKGROUND**
2. What types of research/specific projects have you been working on over the past 3 years or so?

- main aims, scale, where conducted, types of participants, collaborators, funding
- types of data used

1. **SPECIFIC EXPERIENCES OF RESEARCH USING CLINICAL DATA**
2. **General information** on data sharing experiences: Can you talk about a specific example when you used individual anonymized routinely collected clinical or laboratory data to support your research?

- What was the research question and what types of routinely collected clinical or laboratory data were used?
- Where did the data come from? Which institution and what was the clinical background of people whose data were used?
- How did this request come about? E.g. was this a planned form of data access in an existing approved protocol?
- How long after this clinical data was collected was it being used for the research project? (e.g. less/more than 5 years?)

1. In this situation, what was the **process of being given access to clinical data**?

- Who made decisions about whether the data could be given? How?
- **Were there any differences of opinion**? If so, what were these about, **how were they resolved** and what did you think about this process? Could it have been done in a **better way**?
- Were there any forms/agreements/policy documents used? What were these? Are you able to provide copies?
- Do you know how this data sharing system was set up? What are your views on how the system is working in general (Probe: whether stakeholders involved are appropriate/any missing voices; timeliness; transparency; accountability)
- What did you think about the data sharing process you experienced from a practical point of view – what was **straightforward or difficult**? What changes would you like to see, and why?

**USE OF ROUTINELY COLLECTED CLINICAL DATA TO SUPPORT AUDITS**

*I’d now like to ask you some questions that look at patients’ perspectives on different uses of clinical data. We think this area is important because at the moment there are no evidence based policies on the ways that patients should be involved when clinical data is used for a range of different purposes, including routine auditing or reporting, programme evaluations or to support research in different ways. In the rest of this interview I’d like to hear your views on ways in which you think patients or the wider public should be informed or involved, across a number of different scenarios.*

1. To what extent do you think patients admitted to hospitals in Kenya **are** **aware** that their clinical data - without names or other identifiers - are used as part of routine auditing and reporting processes?

- To what extent do you think it’s important for people **to be aware** of this?
- Can you explain **why** do you think this is/is not important? If so, how should this be done?
  - *Probe for reasons seen as important and as difficult/wrong thing to do*
  - *Where see awareness raising as important: Some people have worried that telling patients about this might in some cases lead to people not accepting this use – does this worry you in any way?*
  - *Retrospective/prospective: If interviewee describes challenges of following up patients who have been discharged: remind that, going forwards, explaining this use to patients during admission might make it unnecessary to follow up after discharge*
- *If consider creating awareness is important:* How urgently do you think this should be introduced as a policy?
- Any experiences of this being done, and how did it work out?

1. In some situations, we do more than create awareness but actually ask **permission** (or ask for consent) from patients for certain types of activities to happen. To what extent do you think it would appropriate to ask individual patients for permission (rather than just make sure aware) for their data to be used in these ways?

- Can you explain **why** do you think this is/is not important? [*Compare with views on whether patients should be aware, and find out reasons for differences between these responses. As above, check that considering this prospectively*].
- *If consider creating awareness is important:* As before, how urgently do you think this should be introduced as a policy?
- If so, how should this be done?
- In situations where getting permission from patients is not really possible, what should be done?

1. To what extent do you think that the public should know that patient data is used in this way?

- If seen as important, can you explain why you think this?
- If so, how should this be done?

**USE OF ROUTINELY COLLECTED CLINICAL DATA TO SUPPORT EVALUATIONS**

*Explain: We have talked about audits and to what extent you think people should either be aware or give permission for their clinical information to be used for this. Now I would like to shift a bit and ask you the same kinds of question but about clinical information being used as part of evaluations of hospital services. For example:*

| Scenario A: A public health manager in the County Health Team uses clinical and laboratory data from individual patients who have been treated for malaria in hospital (with names taken off) to evaluate whether new guidelines that have been introduced for the in-patient treatment of malaria are improving clinical outcomes overall and over time. |
| --- |

1. In this situation, compared to the audit (e.g. monthly reports) we were talking about before, do you see any differences in whether patients **should be aware** of this use of their clinical data?

- If seen as different: what do you think should happen? How should this be done?
- And can you explain **why** you think these situations are different in relation to patient awareness?

1. Again, thinking about evaluations, have your views on whether patients **should give permission/consent** changed, compared to the audit situation we were talking about before?

- If seen as different: what do you think should happen, and how?
- And again can you explain why you think these situations are different, in relation to the need for patient consent or permission?
- In situations where getting permission from patients is not really possible, what should be done?
- *If consider creating awareness is important:* How urgently do you think this should be introduced as a policy?

1. What about giving information to the wider **public** about the fact that clinical information is used for programme evaluations – do you feel the same or differently about this for audits/evaluations as for monthly reporting? Why? And how should this be done?
2. *[Based on responses to previous questions*] Does it make any difference to your views about the importance of **awareness**, or the need for **consent** and/or **public awareness** for an evaluation if:

- The evaluation is being done by an NGO that is working with the MOH to implement the programme (not the hospital itself)? *What changes, and can you explain why?*
- In doing this evaluation, the NGO is bringing together information from many different hospitals across the country? *What changes, and can you explain why?*
- The evaluation is being done as a research project by a Kenyan MSc student? Or an international (non Kenyan) researcher? *What changes, and can you explain why?*

**USE OF ‘SENSITIVE’ DATA:**

- Are there any types of data that you think are particularly sensitive and would make a difference to your views so far on whether and how that information could be used for audits or evaluations?
- Can you explain how it would affect your views on the importance of people being aware about uses of their clinical data?
- What about your views on the importance of **asking for consent** for this use?
- Also, what about your views on the need for **public engagement or information giving** about this use?

**RESEARCH USE OF ROUTINELY COLLECTED CLINICAL DATA: NO RANDOMISATION**

*I would now like to ask you some similar questions about how researchers should provide information and/or seek permissions from patients for the use of their clinical data, but now in relation to pragmatic clinical trials. For example:*

| Scenario B: It is common in medical practice that doctors have several different treatments they can use for the same condition, and for it not to be known whether one treatment really works better than another. For example, many different antibiotics are recommended to treat particular infections, like boils, ear infections or lung infections.  In this situation, doctors tend to choose the treatment based on their own or their patients’ personal experiences/preferences. If there was more evidence about which treatments work best and in which situations, both patients and doctors would benefit. One way for researchers to do this is to compare routine clinical data on patient outcomes (e.g. how quickly or completely they got better after being treated by one drug compared to another). In this kind of research, the researchers DON’T introduce anything different to the normal practice. They just analyze the clinical data of patients who were treated to compare the effectiveness of different antibiotics used to treat the infection. |
| --- |

1. **In this situation**: *[Across all section below, include probes on any practical experiences in relation to information sharing or seeking permissions]*

- Does your views about whether patients should be given information about this use of ‘their’ clinical data change compared to the previous situations - where clinical data was being used for audits or when sensitive data was being used for evaluations of services? Should this include asking patients to give permission for this use?
- If different, why do you think it’s different in this situation and not the other? *[Probe: Should give information or ask for permission? Why do you think this is important?]*
- How should hospital management/ researchers make sure they know about /or ask them to give permission for the use of this clinical data? *[Probe for general information e.g. posters or individual explanations and/or consent processes – verbal or written]*
- *Again. where this is different to views expressed around scenarios A to D, probe for reasons behind these differences*

1. **Further probes**: Earlier we talked about whether some changes in the surrounding situation would change your views on whether and how information was shared with individual patients or the wider public. Can we talk about these areas again for this type of research (comparing the effectiveness of different approved treatments)?

- What if that data being collected was about HIV/AIDS? (compare with situation above)
- What if the research was being done by a researcher outside Kilifi:
- A master’s student from a Kenyan university as part of their course requirement?
- A senior researcher from another Kenyan research institute?
- A researcher working in another part of the world including another African country, and outside Africa e.g. Canada?
- What if the findings of the researcher’s work will be published in a science journal that could easily be read by many people?

**RESEARCH USE OF CLINICAL DATA: RCTs**

*The situation we talked about earlier (scenario B) - where researchers assess which of two approved treatments seem to work better by comparing patients - may not always give very good answers. The reason for this is that patients themselves vary in many different ways and that might also affect how well or quickly they recover from an illness.*

*I would now like to introduce a slightly different scenario to the one above. In this case, researchers do not simply use existing clinical data but* ***ALSO*** *use a deliberate process to decide which of the two drugs each patient should be given. This is called a* ***pragmatic randomized controlled trial****.*

Scenario C: There are many different antibiotics currently approved and used routinely for treating pneumonia. For some of these antibiotics, it’s not known if they work better than others available. For example, let’s think about two such treatments, and call them antibiotic X and antibiotic Y. Both are already approved drugs and are in use at the moment. They are given in similar ways and have similar types and risks of any side effects or more serious reactions. (Serious reactions are very rare). It is therefore unlikely that patients or physicians would have a personal preference for one drug over the other. To find out if there are any differences between these treatments, researchers can ask physicians to prescribe one of these drugs based on a system of chance, and observe over time how well patients respond to the treatments. Over time, the outcomes of patients being treated with one of these two antibiotics can be compared to learn which treatment works best. Once this is known, all the patients can be given the option to change to that treatment.

- In this situation, what should patients who are being given one treatment or the other be told?
- Should they be informed about the trial and given a choice about whether to be included, or only be treated as their physician would normally practice, as would be usual in research? Can you explain why you think this is important?
- *[Remind about written informed consent processes] If think should ask for permission:* Should researchers use verbal or written consent processes in this situation? Why/why not?
- What if the research involved additional procedures to those that patients would normally experience? e.g. Filling a questionnaire? More hospital visits for blood sample collection? What difference would this make to your views on information giving to patients and seeking permissions for their involvement?
- What if the trial has been designed so that the physician has the option of using another treatment, other than the one randomly assigned, if they believe doing so is in the best interest of the patient?
- Should the wider group of hospital users/the public be informed that this kind of research activity is taking place in the hospital? Why do you think this? *[Probe for differences with earlier scenarios]* Do you see any difference in how hospital managers should try to inform hospital users in this situation compared to the other situations?

## Clinical Learning Systems project: IDI guide for ward based FWs

*Introduction: In this study, we would like to learn about your views on different ways in which clinical information collected from patients admitted to hospitals like this one are and should be used. I’d like to start by checking that we are both talking about the same thing when we say ‘clinical information or data’.*

1. **Could you let me know what you think ‘clinical information’ is and how it is used?**

*Listen to their explanation, and add on to that to explain: When patients are admitted to the ward, information about them and their illness is primarily collected and used to decide on the best treatment and care for their condition. This is clinical information and can include personal identifiers (their name, address, age etc), their symptoms, what was found on clinical examination, the results of tests done (e.g. blood tests and X rays or scans), the treatment they are given over time and their progress.*

*Explaining use of clinical data for audit: Individual clinical data is also used in other ways, for example, as part of routine audit processes (these are reported to national level also). In these cases, people’s names are removed.*

1. **Are you aware of any forms of audit that uses clinical information in this hospital?**

*Explain what an audit is and how information is used:*

- *An audit is a type of service assessment, to check on how well services are performing against already agreed measures. Audits normally involve collecting data regularly over time from many people.*
- *Examples of types of information that are commonly used in audits are numbers of hospital admission, numbers of cases of malaria, how often recommended drugs are used to treat malaria, whether people recover from their illness and are discharged, and how long they stay in hospital.*
- *Looking at this data overall, health managers can assess whether services are functioning well and which types of services are particularly in demand. They can also learn about patterns of disease and therefore what services are needed. This information is also reported regularly (monthly) to national level to track health issues over the entire country*
- *From this, you can see that clinical information is used in many different ways in addition to ensuring high quality individual patient care for individuals who are admitted to hospital.*
- *For these other uses of clinical data, people’s names and other identifiers (like addresses) are taken off, and the information might be used at the individual level or with data from many individuals brought together.*

1. **TALKING ABOUT AUDITS**
2. To what extent do you think patients are aware that their clinical data - without names or other identifiers - are used as part of routine auditing and reporting processes?

- To what extent do you think it’s important for people to be aware of this?
- Can you explain why do you think this is/is not important? If so, how should this be done?
  - *Probe for reasons seen as important and as difficult/wrong thing to do*
  - *Retrospective/prospective: If interviewee describes challenges of following up patients who have been discharged: remind that, going forwards, explaining this use to patients during admission might make it unnecessary to follow up after discharge*
- *If consider creating awareness is important:* How urgently do you think this should be introduced as a policy?
- Any experiences of this being done, and how did it work out?

1. In some situations, we ask permission (or ask for consent) from patients for certain types of activities to happen. To what extent do you think it would appropriate to ask individual patients for permission (rather than just make sure aware) for their data to be used in these ways?

- Can you explain why do you think this is/is not important? [*Compare with views on whether patients should be aware, and find out reasons for differences between these responses. As above, check that considering this prospectively*].
- *If consider creating awareness is important:* As before, how urgently do you think this should be introduced as a policy?
- If so, how should this be done? Any experiences of this being done, and how did it work out?
- In situations where getting permission from patients is not really possible, what should be done?

1. To what extent do you think that the public should know that patient data is used in this way?

- If seen as important, can you explain why you think this?
- If so, how should this be done?

1. **TALKING ABOUT EVALUATIONS**

*Explain: We have talked about audits and to what extent you think people should either be aware or give permission for their clinical information to be used for this. Now I would like to shift a bit and ask you the same kinds of question but about clinical information being used as part of evaluations of hospital services. Service evaluations aim to assess how well a service is achieving its intended aims. For example:*

| Scenario A: A public health manager in the County Health Team uses clinical and laboratory data from individual patients who have been treated for malaria in hospital (with names taken off) to evaluate whether new guidelines that have been introduced for the in-patient treatment of malaria are improving clinical outcomes overall and over time. |
| --- |

1. In this situation, compared to the audit (e.g. monthly reports) we were talking about before, do you see any differences in whether patients should be aware of this use of their clinical data?

- If seen as different: what do you think should happen? How should this be done?
- And can you explain why you think these situations are different in relation to patient awareness?

1. Again thinking about evaluations, have your views on whether patients should give permission/consent changed, compared to the audit situation we were talking about before?

- If seen as different: what do you think should happen, and how?
- And again can you explain why you think these situations are different, in relation to the need for patient consent or permission?
- In situations where getting permission from patients is not really possible, what should be done?
- *If consider creating awareness is important:* How urgently do you think this should be introduced as a policy?

1. What about giving information to the wider public about the fact that clinical information is used for programme evaluations – do you feel the same or differently about this for audits/evaluations as for monthly reporting? Why? And how should this be done?
2. *[Based on responses to previous questions*] Does it make any difference to your views about the importance of awareness, or the need for consent and/or public awareness for an evaluation if:

- The evaluation is being done by an NGO that is working with the MOH to implement the programme (not the hospital itself)? *What changes, and can you explain why?*
- In doing this evaluation, the NGO is bringing together information from many different hospitals across the country? *What changes, and can you explain why?*
- The evaluation is being done as a research project by a Kenyan MSc student? Or an international (non Kenyan) researcher? *What changes, and can you explain why?*

1. **TALKING ABOUT ‘SENSITIVE’ DATA:**

- Are there any types of data that you think are particularly sensitive and would make a difference to your views so far on whether and how that information could be used for audits or evaluations?
- Can you explain how it would affect your views on the importance of people being aware about uses of their clinical data?
- What about your views on the importance of asking for consent for this use?
- Also, what about your views on the need for public engagement or information giving about this use?

1. ***TALKING ABOUT RESEARCH USE OF CLINICAL DATA: No randomisation***

*I would now like to ask you some similar questions about how researchers should provide information and/or seek permissions from patients for the use of their clinical data, but now in relation to a type of* ***research****. [This type of research is called a pragmatic clinical trial]. For example:*

| Scenario B: It is common in medical practice that doctors have several different treatments they can use for the same condition, and for it not to be known whether one treatment really works better than another. For example, many different antibiotics are recommended to treat particular infections, like boils, ear infections or lung infections.  In this situation, doctors tend to choose the treatment based on their own or their patients’ personal experiences/preferences. If there was more evidence about which treatments work best and in which situations, both patients and doctors would benefit. One way for researchers to do this is to compare routine clinical data on patient outcomes (e.g. how quickly or completely they got better after being treated by one drug compared to another). In this kind of research, the researchers DON’T introduce anything different to the normal practice. They just analyze the clinical data of patients who were treated to compare the effectiveness of different antibiotics used to treat the infection. |
| --- |

1. **In this situation**: *[Across all section below, include probes on any practical experiences in relation to information sharing or seeking permissions]*

- Does your views about whether patients should be given information about this use of ‘their’ clinical data change compared to the previous situations - where clinical data was being used for audits or when sensitive data was being used for evaluations of services? Should this include asking patients to give permission for this use?
- If different, why do you think it’s different in this situation and not the other? *[Probe: Should give information or ask for permission? Why do you think this is important?]*
- How should hospital management/ researchers make sure they know about /or ask them to give permission for the use of this clinical data? *[Probe for general information e.g. posters or individual explanations and/or consent processes – verbal or written]*
- *Again. where this is different to views expressed around scenarios A to D, probe for reasons behind these differences*

1. **Further probes**: Earlier we talked about whether some changes in the surrounding situation would change your views on whether and how information was shared with individual patients or the wider public. Can we talk about these areas again for this type of research (comparing the effectiveness of different approved treatments)?

- What if that data being collected was about HIV/AIDS? (compare with situation above)
- What if the research was being done by a researcher outside Kilifi:
- A master’s student from a Kenyan university as part of their course requirement?
- A senior researcher from another Kenyan research institute?
- A researcher working in another part of the world including another African country, and outside Africa e.g. Canada?
- What if the findings of the researcher’s work will be published in a science journal that could easily be read by many people?

1. **TALKING ABOUT RESEARCH USE OF CLINICAL DATA: RCTs**

*The situation we talked about earlier (scenario B) - where researchers assess which of two approved treatments seem to work better by comparing patients - may not always give very good answers. The reason for this is that patients themselves vary in many different ways and that might also affect how well or quickly they recover from an illness.*

*I would now like to introduce a slightly different scenario to the one above. In this case, researchers do not simply use existing clinical data but* ***ALSO*** *use a deliberate process to decide which of the two drugs each patient should be given. This process is called* ***randomization****. Randomization means choosing something at random, using a system based on chance (e.g. Tossing a coin, and making a choice based on which side the coin landed). Using a random process to choose which of the two treatments a patient is given, and including large numbers of people in the study, will make sure that any individual differences between the people in the study will not affect the final assessment*. [*Use additional explanations for randomization and why its important where needed*]

Scenario C: There are many different antibiotics currently approved and used routinely for treating pneumonia. For some of these antibiotics, it’s not known if they work better than others available. For example, let’s think about two such treatments, and call them antibiotic X and antibiotic Y. Both are already approved drugs and are in use at the moment. They are given in similar ways and have similar types and risks of any side effects or more serious reactions. (Serious reactions are very rare). It is therefore unlikely that patients or physicians would have a personal preference for one drug over the other. To find out if there are any differences between these treatments, researchers can ask physicians to prescribe one of these drugs based on a system of chance, and observe over time how well patients respond to the treatments. Over time, the outcomes of patients being treated with one of these two antibiotics can be compared to learn which treatment works best. Once this is known, all the patients can be given the option to change to that treatment.

- In this situation, what should patients who are being given one treatment or the other be told?
- Should they be informed about the trial and given a choice about whether to be included, or only be treated as their physician would normally practice, as would be usual in research? Can you explain why you think this is important?
- *[Remind about written informed consent processes] If think should ask for permission:* Should researchers use verbal or written consent processes in this situation? Why/why not?
- What if the research involved additional procedures to those that patients would normally experience? e.g. Filling a questionnaire? More hospital visits for blood sample collection? What difference would this make to your views on information giving to patients and seeking permissions for their involvement?
- What if the trial has been designed so that the physician has the option of using another treatment, other than the one randomly assigned, if they believe doing so is in the best interest of the patient?
- Should the wider group of hospital users/the public be informed that this kind of research activity is taking place in the hospital? Why do you think this? *[Probe for differences with earlier scenarios]* Do you see any difference in how hospital managers should try to inform hospital users in this situation compared to the other situations?

## IDI guide for Hospital Management Staff

*In this discussion, I would like to understand your experiences and views around ways that routine clinical data are (or could be) used in ways that are* ***NOT*** *about individual patient care. At present, these data are already used for routine monitoring e.g. in monthly reporting statistics. There are other potential uses for clinical data – with patients’ names taken off and either used individually or aggregated. I would like to get your views on how and whether such uses should be supported and also get your views about the processes that are in place and used to govern the use of clinical data for purposes other than patient care.*

1. **Types of data & their management/storage**
2. How is: a) Clinical data recorded, managed, archived over time? Which departments/ people are involved in this process? b) Laboratory data recorded, managed, archived over time? Which departments/ people are involved in this process?
3. In your experience, what are the strengths and challenges of data management and archiving systems used by your organization?
4. What do you think can be done to improve the management and archiving of the data?
5. **Use of clinical data for audit or evaluation**
6. Are clinical or laboratory data ever used for auditing or evaluating clinical services in this hospital? *[By audits we mean a process that seeks to improve patients care and outcomes through systematic review of care against set standards. Service evaluations on the other hand involves assessing how well a service is achieving its intended aims.]* If so, can you tell me about this? [Probe: How was this done? What data was used, over what period of time and what analyses done? How often does this happen? Who was conducting the audits/ evaluating processes? Whose responsibility is this?] [Ask for access to any auditing or evaluation reports, if possible]

If no known example *or to explore increasing levels of potential controversy*, discuss the following 2 situations:

Clinical hospital data (with people’s names taken off) being used by the County Health Team to assess and report on patterns of different diseases at different times, such as the number of people admitted to hospital with malaria in a given time period.

- To what extent do you think patients are aware that ‘their’ clinical data - without names or other identifiers - are used in these ways?
- Do you think they should be aware? Why do you think this is/is not important?
- If so, how should this be done? [*Probe: including should* ***give information*** *or ask* ***permission****?]* Why do you think this? Any experiences of this being done, and how did it work out?

*[****Probe:*** *When you think about this going forward, should the hospital begin* ***informing*** *patients about this use of clinical data NOW?* ***Why?*** ***How*** *can this be done? What about asking patients for* ***permission*** *NOW going forward? What kind of permission?* ***Why*** *do you think this is important?]*

- Do you think that the **public** should know that patient data is used in this way? Why do you think this is important? How should this be done?

A public health manager in the County Health Team uses clinical and laboratory data from individual patients (with names taken off) to **evaluate whether new guidelines** that have been introduced in different hospitals for clinical care are working well (i.e. improving clinical outcomes).

1. In this situation, do you see any differences in whether patients are aware compared to the monthly routine reporting?

Do you think any differently about whether patients should be aware? If it’s different, why do you think this is important in this situation but not the previous one?

What about information to the public – do you feel the same or differently about this for audits/evaluations as for monthly reporting? Why? And how should this be done? *[Probe:* ***Giving information*** *only or also asking for* ***permission****]* Any experience of this being done, and how did it work out?

1. **Using clinical information to support new research**
2. Are you aware of any instances in the past when clinical or laboratory data in this hospital were used to support research projects that were not directly concerned with auditing or evaluating clinical services? *[Define research as a systematic investigation that seeks to generate generalizable knowledge for the benefit of all people in future]* If so, can you tell me more about that:

- Who was conducting the research, which data were used, what was the research question and what was the outcome of the research?
- Were patients given information about this use of their data? Do you have any measures in place to inform hospital users in general about this use of clinical data? If so, please explain how these systems worked, and how patients/hospital users responded to this information.
- Were patients asked for permission for their anonymized data to be used in this way? If so, how was this done? [Probe: Signed individual informed consent/ general notification]
- *Do you think using clinical data for research and service evaluation initiatives has any implications to public trust and future research? If so, what implications?*

USE CLOSER TO AUDIT: If no awareness of clinical data being used to support research in the past, discuss following scenario:

An **NGO** uses clinical information in aggregated form (without names) collected across a number of different hospitals in Kenya to evaluate the quality of malaria case management, with the aim of improving services in **all hospitals** in Kenya and other similar settings.

- Do you think patients should be aware their clinical data are being used in this situation? Why do you think this is important? [Check for difference in opinion and if so; why do you think this is important in this situation but not the previous one?]
- How should this be done? [Probe: *Should give information or ask permission? Any experiences of this being done, and how did it work out?*]

*[****Probe:*** *When you think about this going forward, should the hospital begin* ***informing*** *patients about this use of clinical data NOW?* ***Why?*** ***How*** *can this be done? What about asking patients for* ***permission*** *NOW going forward? What kind of permission?* ***Why*** *do you think this is important?]*

ALSO:

- In situations where getting permission from patients is not really possible, what should be done?
- We talked earlier about situations in which it might be important to give information about the uses of clinical data in a hospital to the ‘public’ who use that hospital. Do you think this (scenario) is one of those situations? Why do you think this is important here?
- If so, how should this be done? Any experiences of public information/engagement, and how did it work out? *[Probe for general information e.g. posters or individual explanations and/or consent processes]*
- *What if it was a Kenyan Masters student conducting this evaluation? From oversees?*
- *What if the data being collected was about HIV/AIDs? E.g.*

Donors for HIV/AIDS programmes that support service delivery in specialized government outpatient clinics uses clinical and laboratory data collected from clinic users (with names and other identifiers taken off) to support evaluation of the programme they fund.

- In this situation, do you think patients should be aware? Why? Do you see any differences in what information patients should be given or what permissions asked for? What do you think should be done differently, and why do you think this is important in this situation but not in others?
- What about information to the public – do you feel the same or differently about this for sensitive clinical information? Why and what should be done?

1. **Governance of research use of clinical data**: Are there processes in place in your organization to make decisions about what & how clinical data can be used to support new research? If so, what are these? [*e.g. Kilifi DGC*]:

- *How would you describe the data governance structure and how it’s set up in your organization?*
  - Who is involved in making decisions about research use of clinical data? What are the roles and responsibilities of the different stakeholders?
  - How well is this structure monitored to see if it is working? E.g. compliance to policies?
  - How is the data governance system set up to deal with changes over time? (in how data is used, produced and retrieved?) With unexpected events e.g. data breaches?
  - How is clinical and laboratory data collected within the hospital accessible to external requestors? How is access negotiated? What are the challenges to data retrieval and data sharing?
- What do you think are the advantages and disadvantages of these data governance policy/processes? How can they be improved? What would you prefer to see in place (including none)? Why? (if not clear)
- What was the process of setting this system up – who was involved? Should others have been involved? Who?
- Are there any others not involved that you think should be involved, and why? *[Probe for role for patients/public/experts etc.]*

**If there are no such governance processes in place**: please explain what you think would be an ideal governance arrangement, and why. [Probe on how this can be set up, people to be involved and why, why this is important]
